# The Neonatal Assessment Manual scorE: A Reliability Study on Hospitalized Neonates

**DOI:** 10.3389/fped.2021.715091

**Published:** 2021-09-22

**Authors:** Andrea Manzotti, Francesco Cerritelli, Erica Lombardi, Simona La Rocca, Pamela Biasi, Marco Chiera, Matteo Galli, Gianluca Lista

**Affiliations:** ^1^RAISE lab, Foundation COME Collaboration, Pescara, Italy; ^2^Division of Neonatology, Department of Pediatrics, “V. Buzzi” Children's Hospital, ASST-FBF-Sacco, Milan, Italy; ^3^Research Department, SOMA, Istituto Osteopatia Milano, Milan, Italy

**Keywords:** agreement, NAME, newborns, NICU, prematurity, reliability, touch

## Abstract

Despite clinical improvements in neonatal intensive care units (NICUs), prematurity keeps causing several comorbidities. To enhance the management of such conditions, in previous studies we devised the Neonatal Assessment Manual scorE (NAME) model, a structured touch-based assessment that aims to evaluate how newborns respond to gentle touch-based stimuli. The present study aimed to begin assessing the NAME interrater reliability and specific agreements. At the “Vittore Buzzi” Pediatric Hospital NICU ward in Milan, Italy, we enrolled 144 newborns, 85 male and 59 female, with a mean age of 35.9 weeks (±4.1) and a weight of 2,055.3 g (±750.6). Two experienced manual professionals performed the NAME procedure on all the infants. Regarding the total sample and the analysis by sex, we found moderate and statistically significant results for the interrater reliability (*p* < 0.001) and the specific agreements (*p* < 0.05), in particular for the “Marginal” score. Furthermore, interrater reliability significantly (*p* < 0.05) increased as age and weight increased, whereas there was an almost constant moderate and significant (*p* < 0.05) agreement especially for the “Marginal” score. Therefore, we found preliminary results showing that the NAME could be a reliable diagnostic tool for assessing the newborns' general condition.

## Introduction

The improvement in specific medical care helped minimize the chance of pathological complications in neonatal intensive care units (NICUs). The use of advanced technologies in the last decades showed a significant reduction of hospitalization length and mortality rate in the preterm population (1). However, prematurity remains widespread worldwide: it has a global incidence of about 11% but is estimated to be substantially higher in developing countries (2) and brings about several comorbidities including respiratory distress, necrotizing enterocolitis, cardiovascular diseases, neurodevelopmental delay, reduced growth, sepsis, hearing, and visual impairments (3).

To enhance the management of such critical conditions, researchers are investigating all the factors that could improve the premature infants' health: in particular, growing attention has been directed toward touch-related procedures (4, 5). In NICUs, nurses and doctors handle and touch premature babies about a 100 times a day while performing routine-care procedures, including feeding, weighting, applying tubes, changing diapers, performing heel sticks, venipunctures, palliative care procedures, and managing emergencies (4, 6).

Several studies demonstrated that touch could play a central role in perinatal care (5). Different approaches that include gentle touch or similar procedures induced positive effects on the newborns' clinical conditions, such as improved heart rate and oxygen saturation regulation (5, 7–9). Massage therapy, kangaroo care, and osteopathic manipulative treatment also showed positive effects on the survival and growth of preterm babies (8, 10–15). Although these clinical findings give preliminary hints for validating the use of therapeutic touch in NICUs, a procedure that uses touch as an assessment tool is lacking. A manual assessment procedure that is reliable, valid, and easily performed by different NICU professionals would indeed be useful in clinical practice, especially due to the many times infants are already handled. Such manual assessment could integrate with the already existing routine-care touch-based procedures, thus improving infants' care (9).

Different authors tried to include in their evaluations some manual procedures, for instance, the Brazelton Neonatal Behavioral Assessment Scale (BNBAS) and Assessment of Preterm Infants' Behavior (APIB) involve handling or tactile stimulations, but these manual procedures represent only a marginal aspect in a wider behavioral evaluation (16–18). Only one study was published where a touch-based assessment was tested and proposed (19). This study, however, had several limitations regarding the generalization, validity, and sensitivity of the used procedures.

Therefore, a recent model—the Neonatal Assessment Manual scorE (NAME)—was developed aiming to propose a rigorous and structured touch-based evaluation for newborns. It was constructed to be easily used by NICU professionals including physiotherapists, manual therapists, neonatologists, and every other specialist using manual procedures. In particular, the purpose of the NAME is to evaluate how newborns adapt and respond to a gentle touch-based stimulus (9).

As every other evaluation tool, the NAME shall possess both validity and reliability, which are considered the main properties of a diagnostic instrument—they define its practical and clinical usefulness (20). A previous paper has begun assessing the NAME validity (21), whereas the NAME interrater reliability has not been explored yet. Interrater reliability defines the concordance between the measurements obtained by two or more skilled operators. Interrater reliability is, thus, paramount for any diagnostic tool (20, 22).

The objective of the present study was, thus, to begin the evaluation of the NAME interrater reliability and agreement. In addition, we assessed whether interrater reliability and agreement were affected by sex, age, and weight at the time of assessment.

## Materials and Methods

### NAME Description

The NAME model has been developed in the NICU ward, where newborns were assessed in either the incubators or the beds. The NAME procedure consists in applying gentle tactile stimulations on the newborns, having placed one hand on the cranial region with the whole palm and the other hand on the sacral crest with the base phalanges (9). This hands position might change to adapt to the newborns' fragile conditions and the presence of neonatal support devices.

The NAME assessment lasts about 90 s and aims to test the newborns' compliance and homogeneity, i.e., briefly, how the newborns' body mechanically responds to gentle touch through the activation of the autonomic nervous system and the central interoceptive network. In particular, compliance represents whether the body changes its volume according to the applied mechanical stimuli (pressure and distension), whereas homogeneity represents whether the infant's body tissues adapt to the mechanical stimuli in the same way throughout their body [see (9) for in-depth description of the NAME rationale and (21) for an analysis of its validity].

After the evaluation, the operator assigns to the infant a categorical score, which can be one of three possible labels: “Bad” (newborns show neither compliance nor homogeneity), “Marginal” (newborns showed only compliance or homogeneity), and “Good” (newborns show both compliance and homogeneity). Then, the categorical score is converted to a numerical score that consists of a Likert scale ranging from 1 (worst possible score) to 9 (best possible score): in particular, the numerical score ranges from 1 to 3 for “Bad,” from 4 to 6 for “Marginal,” and from 7 to 9 for “Good.” The categorical score, which is the NAME main score, aims to communicate quickly the newborns' actual conditions, whereas the numerical score aims to monitor how the newborns' general conditions change with time.

### Design of the Study

The current study was designed to test the interrater reliability and agreement between two experienced manual therapists performing the NAME assessment.

The experienced manual professionals were identified among a cohort of physiotherapists and osteopaths having more than 10,000 h of clinical practice (23, 24) and specific training in the pediatric field. Two practitioners were recruited and included in the study (age: 42 ± 7, male (%): 1 (50), years of experience: 11.5 ± 7.68).

Operators followed a pre-training phase as suggested in other reliability studies (25, 26). In this research, the pre-training period consisted of 30 h of theoretical overviews, based on the NAME rationale (9), and practical experiences where the operators assessed a total of 50 newborns.

At the end of the pre-training session (the evaluators reached a percentage agreement of 60%), the two operators were identified as Evaluator A and Evaluator B. During the study period, they carried out the NAME procedure on all the enrolled babies, without following a precise examination order to reduce potential biases. Hence, for every baby, one of the two evaluators performed the first NAME procedure avoiding any contact with the other evaluator, who then performed the second NAME procedure 10 min after the first evaluator. This separation time was chosen to wash out any possible influence of the first manual evaluation on newborns. It is worth noting that during this 10-min period the NICU personnel tried not to administer any procedures to the examined infants. In the event that an urgent intervention between evaluations was made, the score was not considered valid.

### Subjects

All newborns entering the “Vittore Buzzi” Pediatric Hospital NICU ward in Milan, Italy, were considered eligible. The study period was from September 2018 to November 2019. The study was approved by the Hospital Ethics Committee (563-04/05/2018) and was conducted according to the Helsinki declaration.

Newborns were excluded if presenting at least one of the following: birth weight <500 g, acute neurological and infectious diseases, major respiratory or cardiovascular instability, undertaken a surgery 2 days before the assessment, born by an HIV-positive or drug-dependent mother, sepsis, and lack of consent to the present study.

One hundred forty-six infants were enrolled; for the whole duration of the present study, they continued the standard medical treatment. The NAME procedure was applied when the infants were asleep or in quiet wakefulness.

### Outcomes

Despite being widely used, reliability measures such as Cohen's k seem to show some critical issues that prevent them from being easily interpretable in clinical settings. In particular, Cohen's k represents a relative measure that relates the observer's measurement to the within-sample variation of the measured parameter, whereas clinicians are usually more interested in the absolute variation of the measured parameter when they perform its evaluation (27, 28).

Cohen's k answers the question “Is it possible to identify abnormalities in a specified sample?” whereas clinicians usually ask “Which is the chance that an operator agrees with the exact measurement I obtained?” This last question requests an absolute measure of percent agreement. Moreover, there could be discordances between Cohen's k values and agreement measures, i.e., the large value of Cohen's k but small value of agreement and vice versa. Hence, Cohen's k may be somehow difficult to interpret and to be clinically meaningful (27, 28). On the contrary, the proportion of specific agreements—the agreements between operators for every specific score of the used scale—could represent an absolute measure with clinical usefulness that answers the question: “Which probability has another operator to agree with my measure/diagnosis?” (28).

Therefore, the present study assessed: (1) the interrater reliability for the numerical NAME scale, whose purpose is not strictly clinical—it serves to monitor the evolution of the newborns' general conditions, thus allowing to recognize “abnormalities” better; and (2) the proportion of specific agreements for the categorical NAME scale, which has more direct clinical usefulness. As pointed out by de Vet and colleagues, the proportion of specific agreements has the potential to give more precise as well as informative data since it measures separately the agreement for every rating of the used scale (28).

Since Cohen's k gives a result concerning the whole assessment scale, the main outcome of the present study was the interrater reliability between two operators for the numerical NAME scale (a Likert scale ranging from 1 to 9).

Secondary outcomes were the following: (1) the proportion of specific agreements for the categorical NAME scale and (2) the interrater reliability and the proportion of specific agreements stratified by sex, age at the time of assessment, and weight at the time of assessment.

## Statistics

### Sample Size Calculation

According to Sim and Wright (29) and Bujang and Baharum (30), we carried out an analysis to estimate the minimum sample size required for Cohen's k. Assuming α = 0.05, β = 0.9, a possible base agreement between observers of 0.3 (regarded as “fair”), and an expected agreement of 0.5 (regarded as “moderate”), we obtained a sample size of 82 participants. Considering the usual 10% increase to account for possible drop-out rate, the total sample size reached 90 infants, which is consistent with this study sample.

### Data Collection and Management

Data were entered into a dedicated data warehouse (31) that was created to record several types of information about the babies, including sex, gestational age, weight at birth, age and weight at the time of NAME assessment, clinical conditions, and side effects.

The data operator was in charge of managing the data warehouse and collecting data into a dedicated *in-house* software, had full access to the system, and did not take part in any clinical activities or manual procedure. The evaluators had access only to the assessment form.

### Statistical Analysis

We checked the collected data for outliers, calculating the mean and standard deviation (SD) for weight and age at the time of NAME assessment: we excluded two subjects whose weight or age lay more than 3 SD from the respective means.

We described the general characteristics of the obtained sample, using mean ± SD: gestational age, weight at birth, age at the time of assessment, and weight at the time of assessment. We reported the number of female and male newborns and described the characteristics stratified by sex.

To explore interrater reliability for the numerical NAME, which is an ordered scale, we performed the weighted Cohen's k test—it takes into account both the disagreement between the two raters and their disagreement degree (32).

The proportion of specific agreements for the categorical NAME was calculated evaluating the actual agreement and the total possible agreement for a given rating (33).

Both these tests were performed on the entire sample, and then the analysis was stratified by sex, weight at assessment, and age at assessment.

The weighted Cohen's k was interpreted according to Landis and Koch (34): ≤0, no agreement; 0.01–0.20, slight agreement; 0.21–0.40, fair agreement; 0.41–0.60, moderate agreement; 0.61–0.80, substantial agreement; and 0.81–1.00, almost perfect agreement. The specific agreement was interpreted based on McHugh et al. considerations about Cohen's k (35): 0–20%, no agreement; 21–40%, minimal agreement; 41–60%, weak agreement; 61–80%, moderate agreement; 81–90%, strong agreement; and 91–100%, almost perfect agreement.

Data were analyzed using the free software R (Version 3.6.1, The R Foundation for Statistical Computing). Statistical significance was set for *p* < 0.05.

## Results

### Sample Characteristics

We enrolled 144 newborns, with a mean gestational age of 32 weeks (±3.6) and a mean weight at birth of 1,667.5 g (±742.5). At the time of the NAME assessment, the newborns were aged 35.9 weeks (±4.1) and had a weight of 2,055.3 g (±750.6). There were 85 (59%) male and 59 (41%) female (Table 1).

**Table 1:** General characteristics of the study population.

	**Female**	**Male**
*N*	59 (41%)	85 (59%)
Gestational age (week)	32.4 ± 3.7	31.7 ± 3.5
Weight at birth (g)	1,636.7 ± 711.6	1,687.1 ± 765.2
Age at assessment (week)	36.4 ± 4.1	35.5 ± 4
Weight at assessment (g)	2,061 ± 807.8	2,051.4 ± 713.1

*Values shown are mean ± SD and N (%)*.

Since the study was carried out in a NICU setting, 126 newborns (87.5%) were preterm, whereas only 18 (12.5%) were full-term. Moreover, Table 2 gives a brief summary of the clinical conditions of all the enrolled newborns.

**Table 2:** Percentage of newborns with certain pathological conditions.

**Pathological conditions**	**Newborns**
Intrauterine growth restriction	40 (27.8%)
Respiratory	79 (54.9%)
Cardiovascular	34 (23.6%)
Gastroenteric	14 (9.7%)
Urogenital	6 (4.2%)
Neurological	6 (4.2%)
Metabolic	54 (37.5%)
Infections	31 (21.5%)
Genetic	9 (6.25%)
Surgical procedures	11 (7.6%)

*Values shown are N (%)*.

### Interrater Reliability and Specific Agreement

Tables 3, 4 summarize the results and interpretations of our main and subgroups analyses. We evaluated the interrater reliability and proportion of specific agreements for the total sample, stratifying by weight and age at assessment.

**Table 3:** Summary of numerical NAME Cohen's k.

	***N***	**Numerical NAME** **Cohen's k**	**k interpretation**
Total sample	144	0.57 (CI: 0.44**–**0.70)^***^	Moderate
Female	59	0.53 (CI: 0.31**–**0.74)^***^	Moderate
Male	85	0.59 (CI: 0.41**–**0.76)^***^	Moderate
**Weight at assessment (g)**			
500–1,499 g	32	0.34 (CI: −0.14 to 0.73)	Fair
1,500–2,499 g	78	0.57 (CI: 0.40**–**0.74)^***^	Moderate
2,500–3,999 g	34	0.79 (CI: 0.63**–**0.94)^***^	Substantial
**Age at assessment (weeks)**			
27–30 weeks	9	0.26 (CI: −0.21 to 0.74)	Fair
31–34 weeks	53	0.35 (CI: 0.04**–**0.65)^*^	Fair
35–38 weeks	47	0.66 (CI: 0.48**–**0.84)^***^	Substantial
39–42 weeks	29	0.74 (CI: 0.57**–**0.92)^***^	Substantial
43–51 weeks	6	0.88 (CI: 0.72**–**1)^***^	Almost perfect

*NAME, Neonatal Assessment Manual scorE; CI, confidence interval*.

* *p < 0.05*.

** *p < 0.01*.

*** *p < 0.001*.

**Table 4:** Summary of categorical NAME proportion of specific agreements.

	***N***	**Categorical NAME agreement**	**Agreement** **interpretation**
Total sample	144	Bad: 0.63 (CI: 0.54**–**0.72)^*^	Moderate
		Marginal: 0.70 (CI: 0.64**–**0.77)^***^	Moderate
		Good: 0.52 (CI: 0.33**–**0.71)	Weak
Female	59	Bad: 0.63 (CI: 0.47**–**0.79)	Moderate
		Marginal: 0.67 (CI: 0.55**–**0.78)^**^	Moderate
		Good: 0.40 (CI: 0.17**–**0.63)	Minimal
Male	85	Bad: 0.63 (CI: 0.51**–**0.74)^*^	Moderate
		Marginal: 0.73 (CI: 0.65**–**0.81)^***^	Moderate
		Good: 0.86 (CI: 0.62**–**1)^**^	Strong
**Weight at assessment (g)**			
500–1,499 g	32	Bad: 0.62 (CI: 0.45**–**0.79)	Moderate
		Marginal: 0.69 (CI: 0.53**–**0.84)^*^	Moderate
		Good: 0.67 (CI: 0.15**–**0.1)	Moderate
1,500–2,499 g	78	Bad: 0.60 (CI: 0.47**–**0.73)	Weak
		Marginal: 0.67 (CI: 0.58**–**0.77)^**^	Moderate
		Good: 0.50 (CI: 0.27**–**0.73)	Weak
2,500–3,999 g	34	Bad: 0.70 (CI: 0.52**–**0.88)^*^	Moderate
		Marginal: 0.78 (CI: 0.66**–**0.90)^***^	Moderate
		Good: 0.50 (CI: 0**–**1)	Weak
**Age at assessment (weeks)**			
27–30 weeks	9	Bad: 0.50 (CI: 0.14**–**0.86)	Weak
		Marginal: 0.60 (CI: 0.30**–**0.90)	Weak
		Good: NA	NA
31–34 weeks	53	Bad: 0.60 (CI: 0.45**–**0.75)	Weak
		Marginal: 0.74 (CI: 0.63**–**0.84)^***^	Moderate
		Good: 0.67 (CI: 0.37**–**0.97)	Moderate
35–38 weeks	47	Bad: 0.67 (CI: 0.50**–**0.83)^*^	Moderate
		Marginal: 0.71 (CI: 0.59**–**0.83)^**^	Moderate
		Good: 0.62 (CI: 0.35**–**0.88)	Moderate
39–42 weeks	29	Bad: 0.62 (CI: 0.39**–**0.86)	Moderate
		Marginal: 0.74 (CI: 0.60**–**0.87)^**^	Moderate
		Good: 0 (CI: 0**–**0)	None
43–51 weeks	6	Bad: 0.75 (CI: 0.47**–**1)	Moderate
		Marginal: 0 (CI: 0**–**0)	None
		Good: 0 (CI: 0**–**0)	None

*NAME, Neonatal Assessment Manual scorE; CI, confidence interval; NA, no subject received the corresponding score*.

* *p < 0.05*.

** *p < 0.01*.

*** *p < 0.001*.

Regarding the total sample, the interrater reliability for the numerical NAME [0.57, confidence interval (CI) 0.44–0.70] and the specific agreements for the categorical NAME “Bad” (0.63, CI 0.54–0.72) and “Marginal” (0.7, CI 0.64–0.77) scores were moderate and statistically significant (*p* < 0.05 for the “Bad” score and *p* < 0.001 for the interrater reliability and the “Marginal” score).

**Figure 1:**
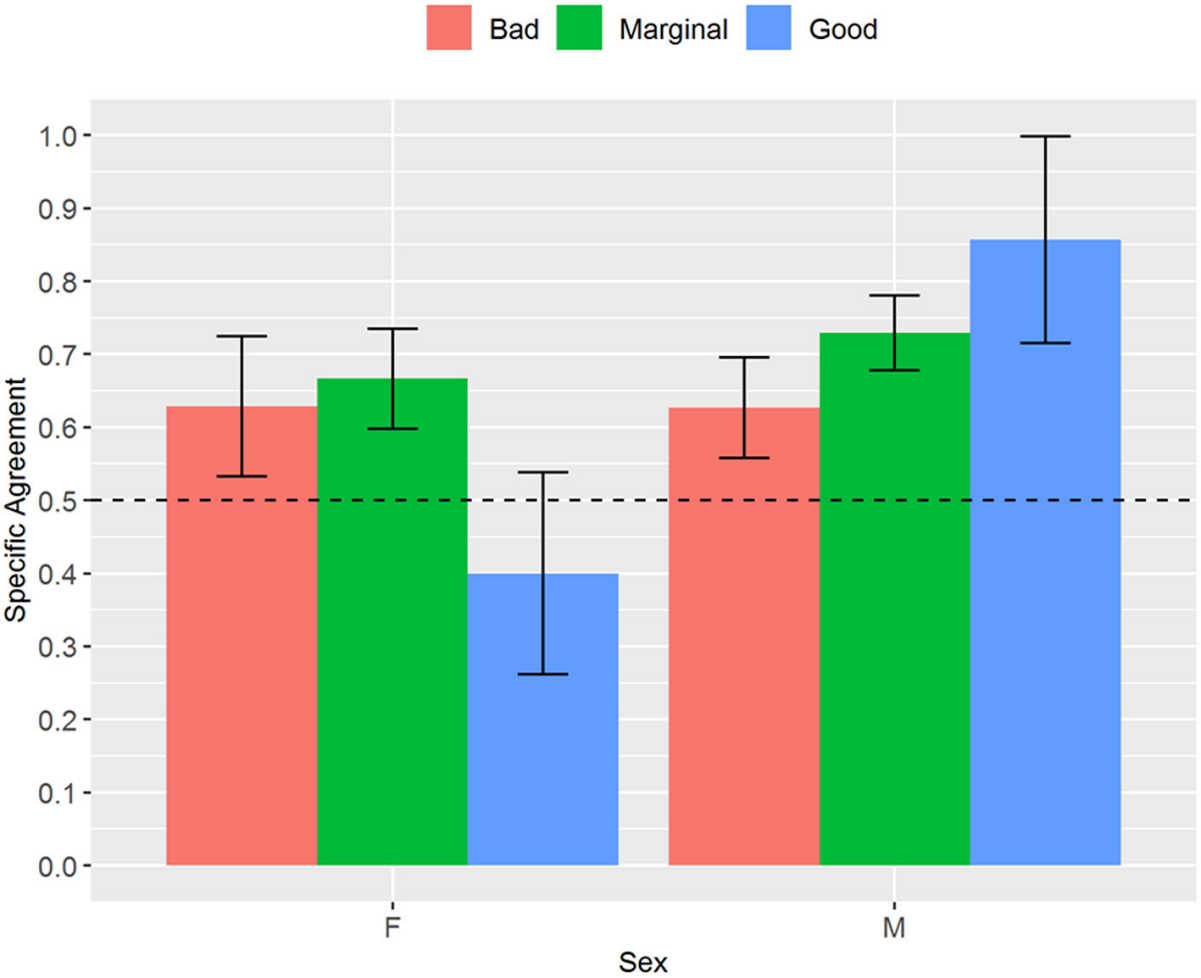
Proportion of specific agreements for categorical NAME grouped by sex. NAME, Neonatal Assessment Manual scorE.

Considering the analysis stratified by sex, we found a moderate and statistically significant (*p* < 0.001) interrater reliability for the numerical NAME for both female (0.53, CI 0.31–0.74) and male newborns (0.59, CI 0.41–0.76), but the specific agreements for the categorical NAME differed between the two subgroups. All the specific agreements for male newborns reached statistical significance (*p* < 0.05) and were at least moderate (Bad: 0.63, CI 0.51–0.74; Marginal: 0.73, CI 0.65–0.81; Good: 0.86, CI 0.62–1), while for the female newborns only the specific agreement for the “Marginal” score reached statistical significance (0.67, CI 0.55–0.78, *p* < 0.01) (Figure 1).

**Figure 2:**
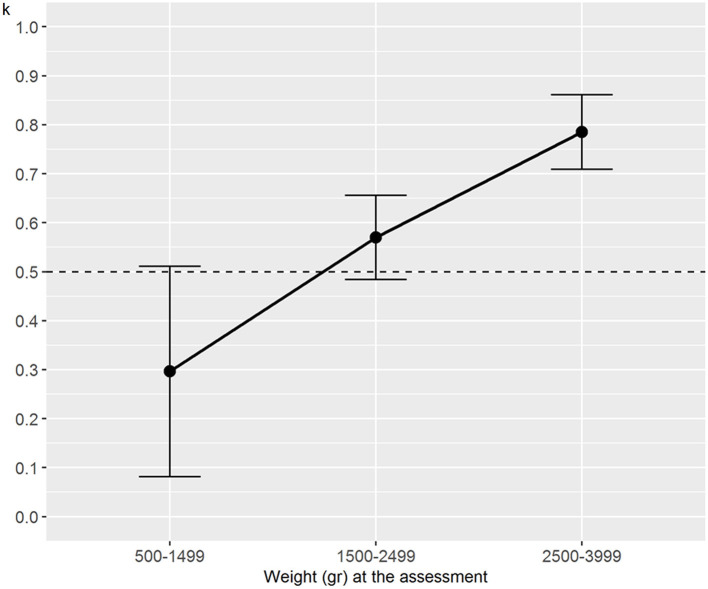
Weighted Cohen's k for numerical NAME grouped by weight at assessment. NAME, Neonatal Assessment Manual scorE.

**Figure 3:**
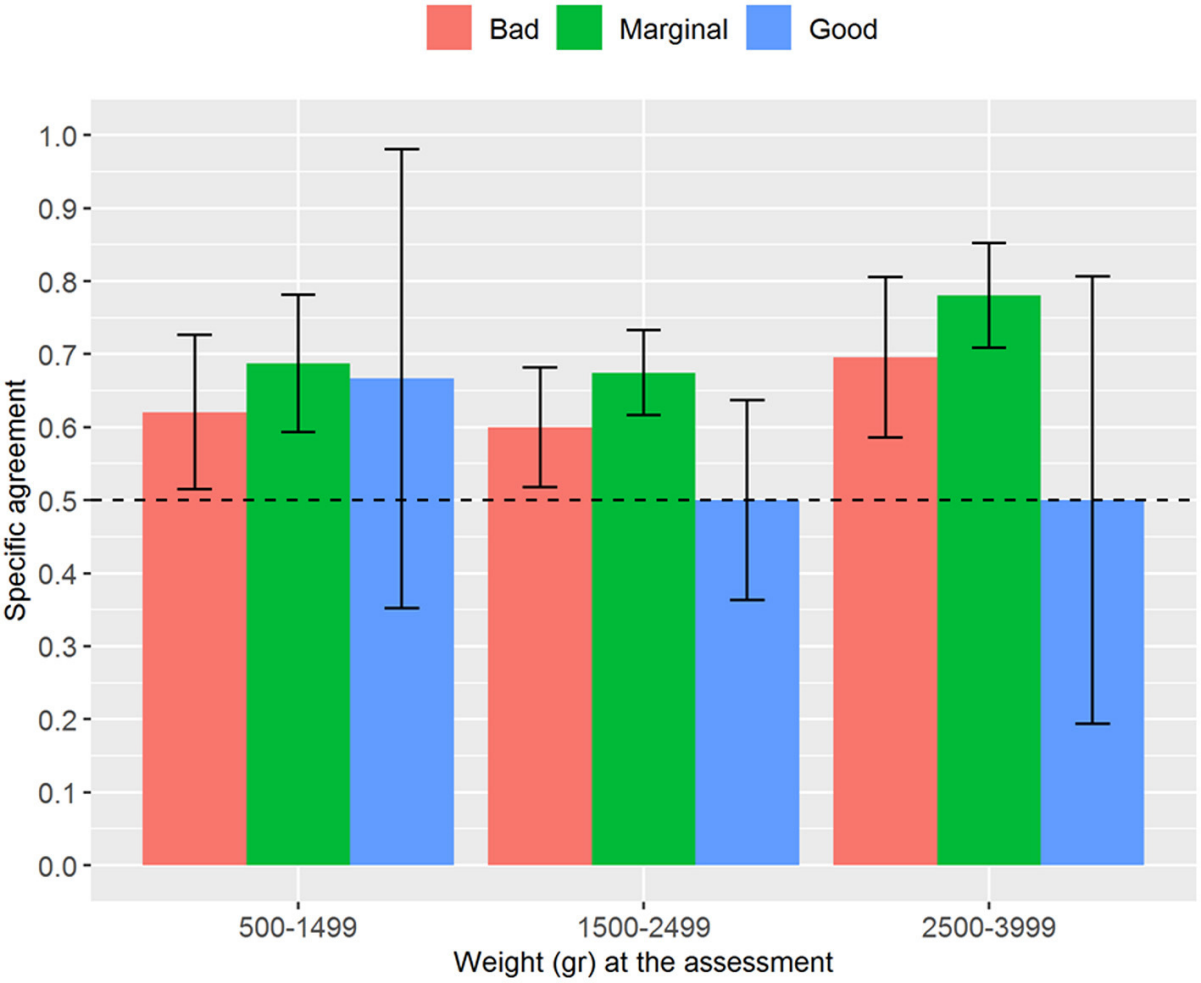
Proportion of specific agreements for categorical NAME grouped by weight at assessment. NAME, Neonatal Assessment Manual scorE.

Regarding the analysis stratified by weight, the interrater reliability for the numerical NAME reached statistical significance (*p* < 0.001) for the groups 1,500–2,499 and 2,500–3,999 g and tended to increase with the newborns' weight—it passed from fair (weight between 500 and 1,499 g) to substantial (weight between 2,500 and 3,999 g) (Figure 2). We found generally moderate specific agreements for the categorical NAME, but statistical significance (*p* < 0.03) was reached almost only for the “Marginal” score (Figure 3). Supplementary analyses were conducted by stratifying the sample differently in order to further test the reliability parameters (Supplementary Tables 1, 2, Supplementary Figures 1, 2). Results confirmed the main analysis but showed that all the categories reached statistically significant weighted Cohen's k values apart from the 1,000–1,499 g group.

**Figure 4:**
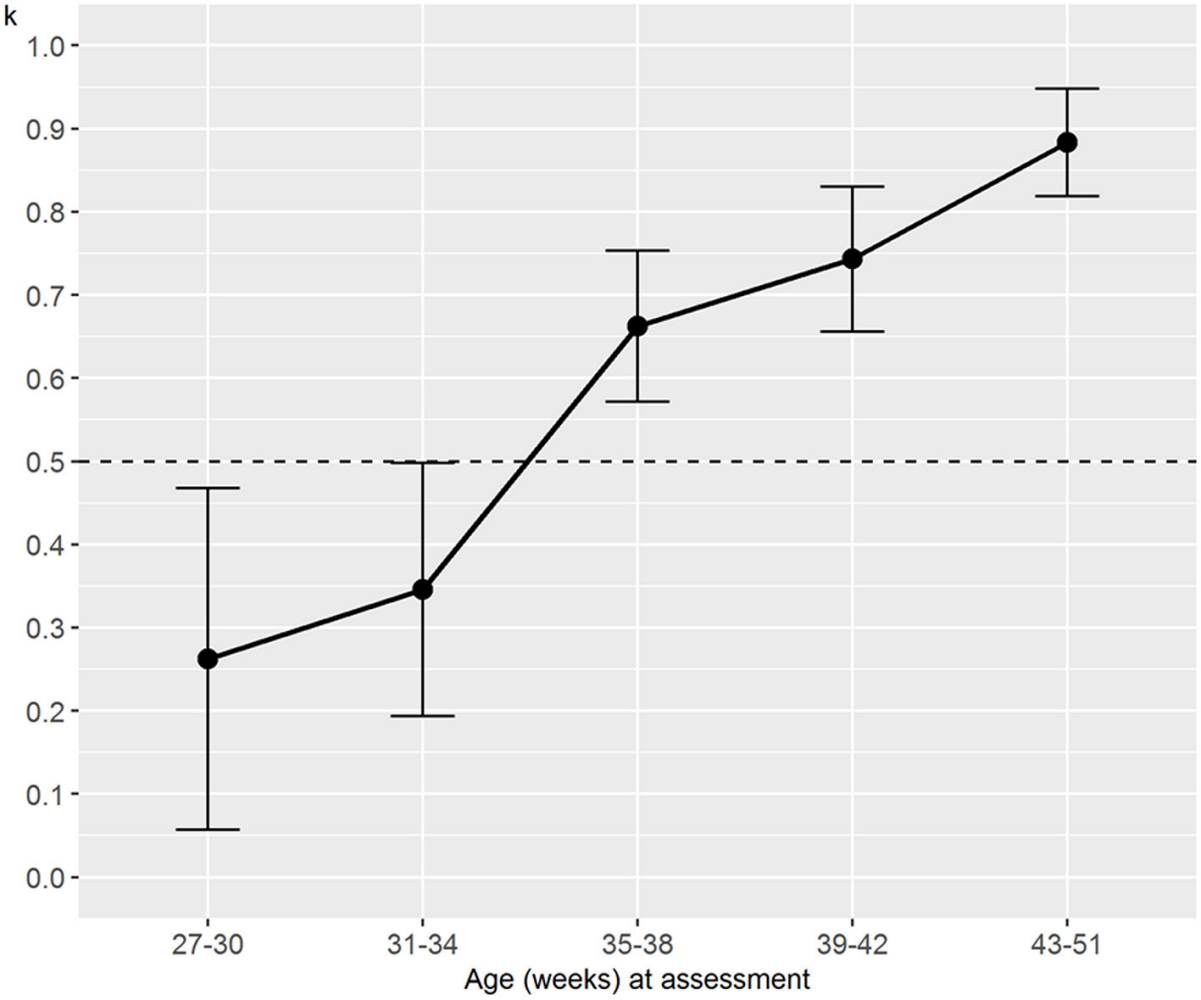
Weighted Cohen's k for numerical NAME grouped by age at the assessment. NAME, Neonatal Assessment Manual scorE.

**Figure 5:**
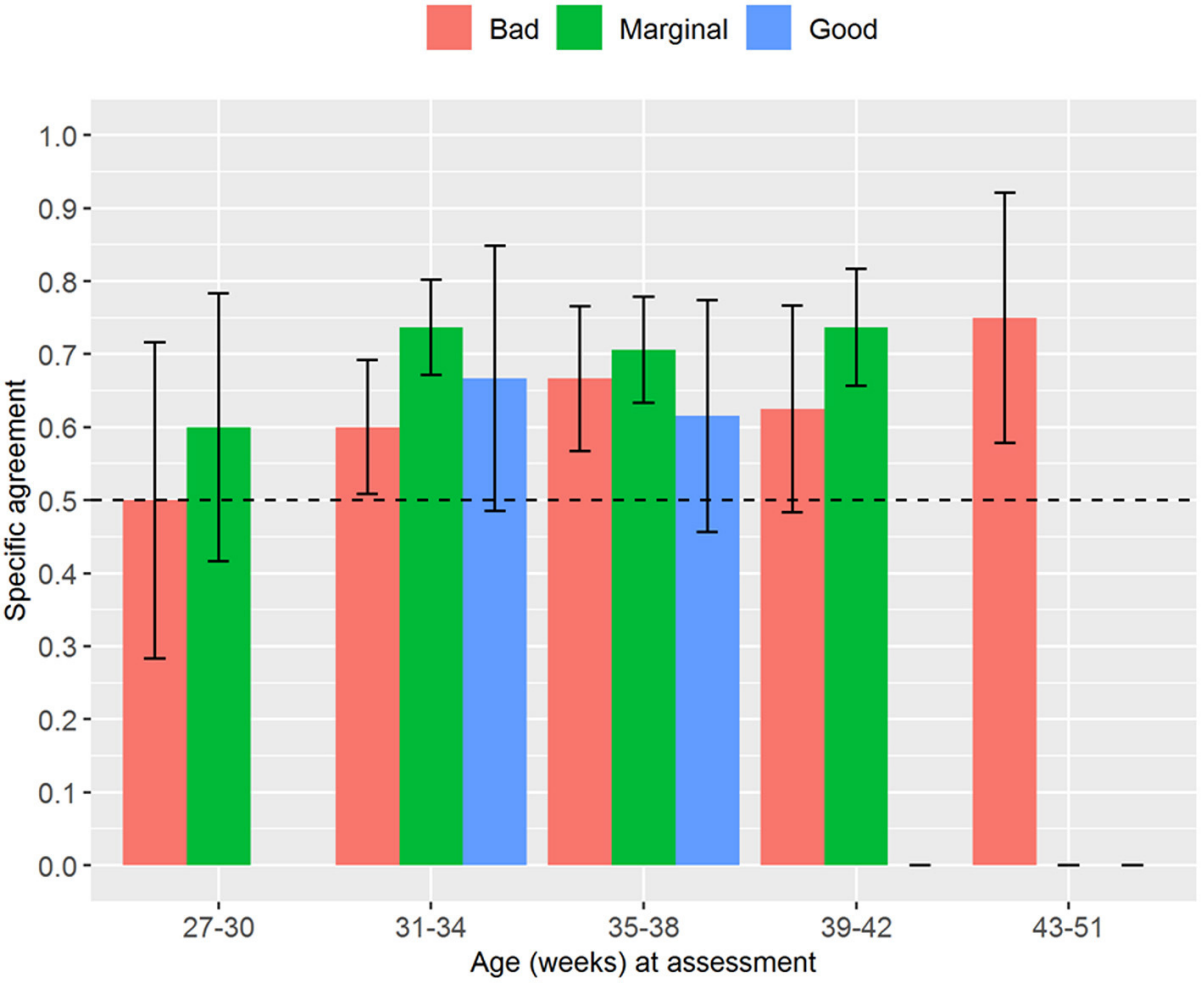
Proportion of specific agreements for categorical NAME grouped by age at the assessment. No one received a “Good” score in the group 27–30 weeks. NAME, Neonatal Assessment Manual scorE.

Regarding the analysis stratified by age, we found trends similar to the analysis stratified by weight. The interrater reliability for the numerical NAME increased with the newborns' age, and it always reached statistical significance (*p* < 0.03) except for the age between 27 and 30 weeks (Figure 4). The specific agreements for the categorical NAME were generally moderate and again reached statistical significance (*p* < 0.05) almost only for the “Marginal” score (Figure 5).

## Discussion

Several studies demonstrated that procedures involving gentle touch, including light massage, osteopathic manipulative treatment, and kangaroo care, can induce positive effects in preterm babies: lower stress levels, better sleep quality, augmented heart rate and oxygen saturation regulation, improved growth, and higher survival likelihood (5, 7–15). However, since a procedure that uses touch purely as an assessment tool is lacking, we devised the NAME procedure: we described its rationale (9), started its validation process (21), and, in the present study, assessed its reliability.

We found a moderate interrater reliability for the NAME numerical scale and moderate specific agreements for the “Bad” and “Marginal” scores of the NAME categorical scale. These results seem to be consistent for both the total sample and the stratification analysis in male and female infants. Concerning the stratification by age and weight at the time of assessment, we found that interrater reliability for the numerical NAME increases as the age and weight at the assessment increase, whereas the categorical NAME shows, despite some exceptions, an almost constant moderate proportion of specific agreement across every interval.

Addressing the obtained results more thoroughly, it is worth noting the difference we found between female and male preterms using the categorical NAME: indeed, there was a strong agreement for the “Good” score in male but not in female preterms, which, on the contrary, showed a minimal agreement for the “Good” score. Several studies point out that preterm males and females display a disparity in disease outcomes: in particular, premature males show a higher mortality rate, a higher susceptibility to infections, and a more immature immune system than premature females (36–39). These differences could make the more stable and healthy male infants to be more easily recognizable as “Good” than female infants who seem to show instead more complex and non-linear physiological regulation (40, 41). Nonetheless, our results require further research to evaluate specifically this sex effect.

In regards to age and weight, since they seem to correlate with the morbidity and mortality in the infants, these variables could have a direct influence on the babies' health and behavioral complexity (42–44). Hence, we might suppose that age and weight could also affect the NAME reliability: potentially, the NAME could be more reliable in infants displaying better health conditions. This could also be due to the fact that the NAME reserves more levels to categorize potentially problematic infants: indeed, across the 1–9 Likert scale for the numerical NAME, only three values (from 7 to 9) define “Good” infants displaying both compliance and homogeneity.

Therefore, the results we obtained about the categorical NAME scale, which represent the more clinically useful score (9), become paramount to better comprehend the NAME usefulness as a diagnostic tool. Indeed, we calculated the proportion of specific agreements for the categorical NAME scale because, according to some authors, Cohen's k does not provide useful information for clinical practice (27, 28). As already stated, our results show a moderate agreement computed on the total sample for both the “Marginal” and “Bad.” With few exceptions due to small sample size, we also found a level of agreement at least moderate for the “Marginal” and “Bad” categories across all the age and weight intervals. In this stratified analysis, the “Marginal” score was the one that resulted as statistically significant most of the time: this result could be due to both the newborns' conditions—it is less likely that infants are in “Good” conditions, otherwise they would not be hospitalized in NICUs, and in the same way it is less likely that infants are in “Bad” conditions since NICU professionals are taking care of them—and the small sample size of the subgroups.

Since to the best of our knowledge there are no studies that have used the proportion of specific agreements to evaluate the reproducibility of a diagnostic test in NICUs, we might consider the moderate agreement we obtained as a preliminary satisfying result for the categorical NAME. More importantly, this positive result could prove that the categorical NAME scale represents a more affordable and clinically useful scale than the numerical one: indeed, the proportion of specific agreements remained almost constant across every age and weight interval.

To understand whether the NAME can be useful in neonatology, it is paramount to compare it with the clinical tools that are widely used to evaluate babies. One example is the Test of Infant Motor Performance (TIMP), which is a structured visual assessment of posture and movement control that aims to assess both spontaneous and elicited motor behavior in infants under 4 months of age (45). Finkel and colleagues tested the interrater reliability of TIMP between two evaluators and found a weighted k of 0.61 (46). Our results regarding the numerical NAME were similar to the one from the study by Finkel et al. (respectively, 0.57 vs. 0.61), despite differences in the sample type, the size of the population enrolled, and the performed manual assessment. Moreover, unlike the TIMP and similar scales, which rely minimally on manual evaluation—it is limited just to testing muscle tone or general behavior—the NAME is a diagnostic tool that focuses on manual evaluation.

However, after a closer look at the data of Finkel et al., their use of the weighted k seems to be inappropriate since this statistic requires only two evaluators to be involved in the procedure (46). When a limited number of operators is chosen from a wider population of operators, as in the study by Finkel et al. (46), the intraclass correlation coefficient (ICC) seems to be a more appropriate index to be used (although other statistics could be chosen). In particular, the authors could have used the ICC (1, 1) statistic since each subject was measured by a different set of two selected operators. Therefore, it seems quite difficult to properly compare our results with those of Finkel and colleagues to see whether the NAME and the TIMP share the same level of reliability (29, 35, 47).

Furthermore, although the TIMP is recognized as safe and well-tolerated by preterm infants, in the trial by Finkel et al. the TIMP proved stressful due to its time length and the positions the infants were to tolerate (46). Despite the fact that the study has been conducted on infants with spinal muscular atrophy type I, who constitute a delicate population that could experience stress more easily, our study on the NAME did not find any stressful responses expressed by the infants, no matter their health condition.

A limitation of the present study is the difference between the sample sizes of the subgroups obtained by stratifying the analysis by sex, age, and weight. This limitation could have impacted on the interrater reliability and specific agreements, in particular, on the mixed results we discussed above. Nonetheless, the small sample sizes obtained in the subgroups reflect the clinical reality of the NICU where we carried out the present study: therefore, this result could encourage researchers to perform further studies focused on these subgroups separately.

Another limitation lies in the difficult comparison between the NAME model and other evaluation models, given that the NAME is the first procedure that involves only a manual evaluation of the newborns. The lack of a gold standard, which we had to face in the NAME development and validity assessment, was partially overcome by defining a rationale based on the best available evidence about the effects of touch on newborns and the neurological regulation of the newborns' body, and on the clinical usefulness that this evaluation tool will bring to NICU professionals (9).

The NAME procedure could also have been biased by infant care; that is, the evaluators could have been induced to give infants a “Good” or “Bad” score based, for instance, on the technologies used to take care of them [e.g., nasal continuous positive airway pressure (nCPAP), mechanical ventilation]. Indeed, as highlighted in the paper where we have defined the NAME rationale (9), the haptic perception used to carry out the NAME procedure can be affected by top–down cognitive processes regarding the supposed clinical conditions of the newborns. For this reason, the operators decided to obtain information concerning the medical history of the examined newborns only after the assessment, thus trying to reduce this kind of bias the best as they could.

A more relevant limitation of the present study is the effect that the NAME procedure itself can have on the scores given by the evaluators due to the effects of touch. In fact, many studies have shown that gentle touch can improve the infants' clinical conditions (5, 7–15). Therefore, it is likely that just placing two hands on the infants and applying little pressure, as it happens during the NAME procedure, could affect the babies' conditions, through the activation of C-tactile fibers, Merkel-neurite complexes, and maybe other pathways (9). In particular, in the present study, the first evaluator could induce an effect that could last until the second evaluator performs the NAME, thus biasing the second score. Past studies evaluated the effects of touch only for 5 min after having applied gentle touch procedures to the babies (5, 8); hence, we do not know whether those effects could last for 10 min (the wash-out period followed by the two evaluators) or even more. Surely, future research about the NAME should assess whether the NAME itself might represent a sort of “therapeutic intervention” due to the intrinsic effects of touch.

In fact, the present study evaluated the interrater reliability and agreement of the NAME, as a part of the validation process of the NAME procedure. According to a previous study, the NAME seems to have both face and content validity; we also found some preliminary positive results for the NAME construct validity by assessing the relationships between age, weight, and the NAME (21). To strengthen the NAME validity, it is also necessary to evaluate whether there is a relationship between the NAME and the newborns' clinical conditions (for instance, the conditions reported in Table 2). This analysis will allow us to characterize better the NAME construct validity, that is, whether the test measures the concept that it is intended to measure (22), and to effectively realize the NAME clinical usefulness for NICU operators.

## Conclusion

The present paper found preliminary results that the NAME could be a reliable diagnostic tool for assessing the newborns' general condition: in particular, the categorical NAME could indeed be more reliable and, thus, clinically useful than the numerical one.

As a previous study started to assess the NAME validity and found preliminary results about its construct validity, future studies should evaluate the relationship between the NAME and the infants' clinical conditions to strengthen both the NAME construct validity and its clinical usefulness in the NICU setting. Furthermore, it could also add other clues about the different usefulness of the categorical and the numerical scales.

Since the NAME procedure represents the only diagnostic assessment for newborns based entirely on manual stimuli, the results obtained through these studies could also contribute to the growth of the manual therapies profession in a multidisciplinary hospital context.

## Data Availability Statement

The original contributions presented in the study are included in the article/Supplementary Material, further inquiries can be directed to the corresponding author/s.

## Ethics Statement

The studies involving human participants were reviewed and approved by Buzzi Hospital Ethics Committee (563-04/05/2018). Written informed consent to participate in this study was provided by the participants' legal guardian/next of kin.

## Author Contributions

AM, MG, EL, SL, and PB: conceptualization, methodology, and writing–original draft preparation. FC and MC: formal analysis. AM, EL, SL, and PB: investigation. AM, MG, EL, SL, PB, FC, MC, and GL: writing–review and editing. GL: supervision. All authors approved the final manuscript as submitted and agree to be accountable for all aspects of the work.

## Conflict of Interest

The authors declare that the research was conducted in the absence of any commercial or financial relationships that could be construed as a potential conflict of interest.

## Publisher's Note

All claims expressed in this article are solely those of the authors and do not necessarily represent those of their affiliated organizations, or those of the publisher, the editors and the reviewers. Any product that may be evaluated in this article, or claim that may be made by its manufacturer, is not guaranteed or endorsed by the publisher.
