# Supplementary material for: The Neonatal Assessment Manual scorE: A Reliability Study on Hospitalized Neonates
Source: Front Pediatr. 2021 Sep 22;9:715091. doi: 10.3389/fped.2021.715091 (PMC8492991; doi:10.3389/fped.2021.715091)
Supplement: Supplementary file 1 [file Data_Sheet_1.docx]

**Supplementary Materials:** The following tables and figures show a more in-depth stratification of the total sample by weight and age at the time of assessment: every subgroup is accompanied by the correspondent weighted Cohen’s k and percentage of specific agreement.

**Table S1.** Summary of numerical NAME weighted Cohen’s k stratified by weight and age at assessment

|  | **N** | **Numerical NAME Cohen's k** | **k Interpretation** |
| --- | --- | --- | --- |
| *Weight at assessment (gr)* | | | |
| **500-999 gr** | 5 | 0.34 (CI: 0.01-0.67)^§^ | Fair |
| **1000-1499 gr** | 27 | 0.3 (CI: -0.15-0.75) | Fair |
| **1500-1999 gr** | 52 | 0.42 (CI: 0.18-0.66)^†^ | Moderate |
| **2000-2499 gr** | 26 | 0.81 (CI: 0.65-0.97)^†^ | Almost perfect |
| **2500-2999 gr** | 10 | 0.69 (CI: 0.36-1)^†^ | Substantial |
| **3000-3499 gr** | 16 | 0.67 (CI: 0.36-0.98)^†^ | Substantial |
| **3500-3999 gr** | 8 | 0.94 (CI: 0.83-1)^†^ | Almost perfect |
| *Age at assessment (weeks)* | | | |
| **27-30 weeks** | 9 | 0.26 (CI: -0.21-0.74) | Fair |
| **31-34 weeks** | 53 | 0.35 (CI: 0.04-0.65)* | Fair |
| **35-38 weeks** | 47 | 0.66 (CI: 0.48-0.84)^†^ | Substantial |
| **39-42 weeks** | 29 | 0.74 (CI: 0.57-0.92)^†^ | Substantial |
| **43-46 weeks** | 4 | 0.92 (CI: 0.77-1)^†^ | Almost perfect |
| **47-50 weeks** | 1 | NA (CI: NA-NA) | NA |
| **51-54 weeks** | 1 | NA (CI: NA-NA) | NA |

^1^ NA: not enough subjects to calculate weighted Cohen’s k

^*^ p < 0.05

^§^ p < 0.01

^†^ p < 0.001

**Table S2.** Summary of categorical NAME proportion of specific agreements stratified by weight and age at assessment

|  | **N** | **Categorical NAME Agreement** | **Agreement Interpretation** |
| --- | --- | --- | --- |
| *Weight at assessment (gr)* | | | |
| **500-999 gr** |  | Bad: 0.57 (CI: 0.2-0.94) | Weak |
|  | 5 | Marginal: 0 (CI: 0-0) | None |
|  |  | Good: NA | NA |
| **1000-1499 gr** |  | Bad: 0.64 (CI: 0.44-0.83) | Moderate |
|  | 27 | Marginal: 0.76 (CI: 0.61-0.9)^§^ | Moderate |
|  |  | Good: 0.67 (CI: 0.15-1) | Moderate |
| **1500-1999 gr** |  | Bad: 0.59 (CI: 0.42-0.75) | Weak |
|  | 52 | Marginal: 0.68 (CI: 0.56-0.79)^§^ | Moderate |
|  |  | Good: 0.36 (CI: 0.06-0.67) | Minimal |
| **2000-2499 gr** |  | Bad: 0.62 (CI: 0.39-0.86) | Moderate |
|  | 26 | Marginal: 0.67 (CI: 0.49-0.84) | Moderate |
|  |  | Good: 0.67 (CI: 0.37-0.97) | Moderate |
| **2500-2999 gr** |  | Bad: 0.86 (CI: 0.62-1)^§^ | Strong |
|  | 10 | Marginal: 0.92 (CI: 0.8-1)^†^ | Almost perfect |
|  |  | Good: NA | NA |
| **3000-3499 gr** |  | Bad: 0.6 (CI: 0.3-0.9) | Weak |
|  | 16 | Marginal: 0.67 (CI: 0.46-0.88) | Moderate |
|  |  | Good: 0.5 (CI: 0-1) | Weak |
| **3500-3999 gr** |  | Bad: 0.67 (CI: 0.3-1) | Moderate |
|  | 8 | Marginal: 0.8 (CI: 0.57-1)* | Moderate |
|  |  | Good: NA | NA |
| *Age at assessment (weeks)* | | | |
| **27-30 weeks** |  | Bad: 0.5 (CI: 0.14-0.86) | Weak |
|  | 9 | Marginal: 0.6 (CI: 0.3-0.9) | Weak |
|  |  | Good: NA | NA |
| **31-34 weeks** |  | Bad: 0.6 (CI: 0.45-0.75) | Weak |
|  | 53 | Marginal: 0.74 (CI: 0.63-0.84)^†^ | Moderate |
|  |  | Good: 0.67 (CI: 0.37-0.97) | Moderate |
| **35-38 weeks** |  | Bad: 0.67 (CI: 0.5-0.83)* | Moderate |
|  | 47 | Marginal: 0.71 (CI: 0.59-0.83)^§^ | Moderate |
|  |  | Good: 0.62 (CI: 0.35-0.88) | Moderate |
| **39-42 weeks** |  | Bad: 0.62 (CI: 0.39-0.86) | Moderate |
|  | 29 | Marginal: 0.74 (CI: 0.6-0.87)^§^ | Moderate |
|  |  | Good: 0 (CI: 0-0) | None |
| **43-46 weeks** |  | Bad: 0.8 (CI: 0.48-1) | Moderate |
|  | 4 | Marginal: 0 (CI: 0-0) | None |
|  |  | Good: 0 (CI: 0-0) | None |
| **47-50 weeks** |  | Bad: 1 (CI: 1-1)^†^ | Almost perfect |
|  | 1 | Marginal: NA | NA |
|  |  | Good: NA | NA |
| **51-54 weeks** |  | Bad: 0 (CI: 0-0) | None |
|  | 1 | Marginal: 0 (CI: 0-0) | None |
|  |  | Good: NA | NA |

^1^ NA: no subject received the corresponding score

^*^ p < 0.05

^§^ p < 0.01

^†^ p < 0.001


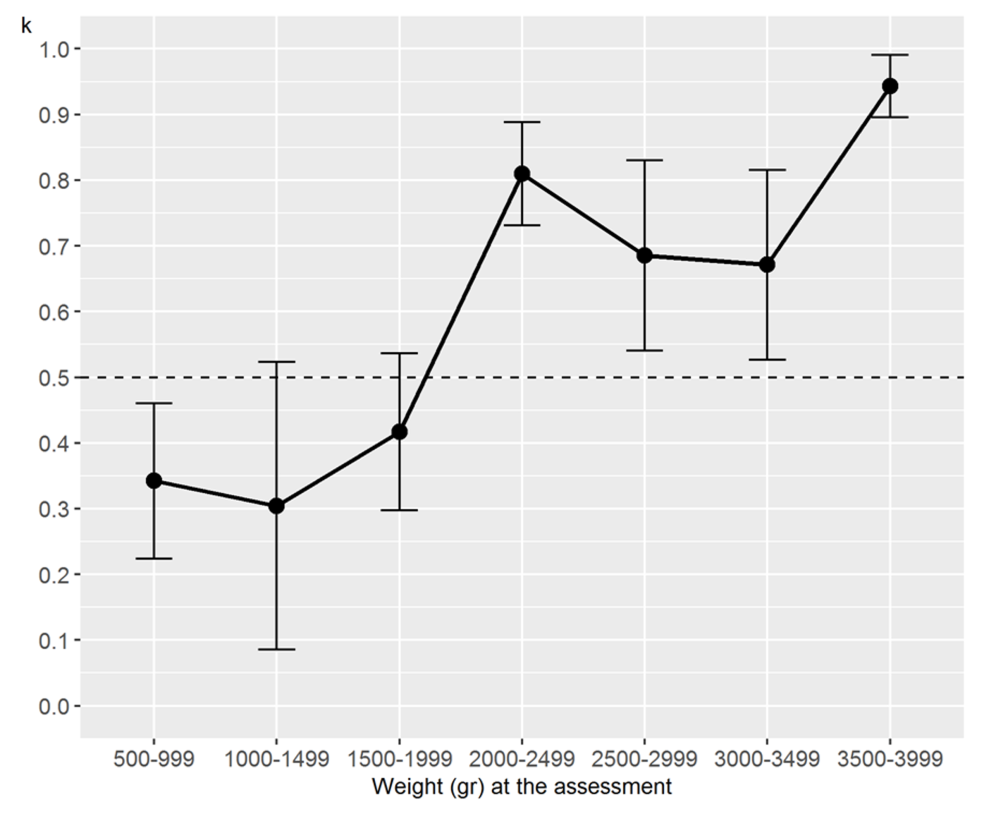


**Figure S1.** Weighted Cohen's k for numerical NAME grouped by weight at assessment.


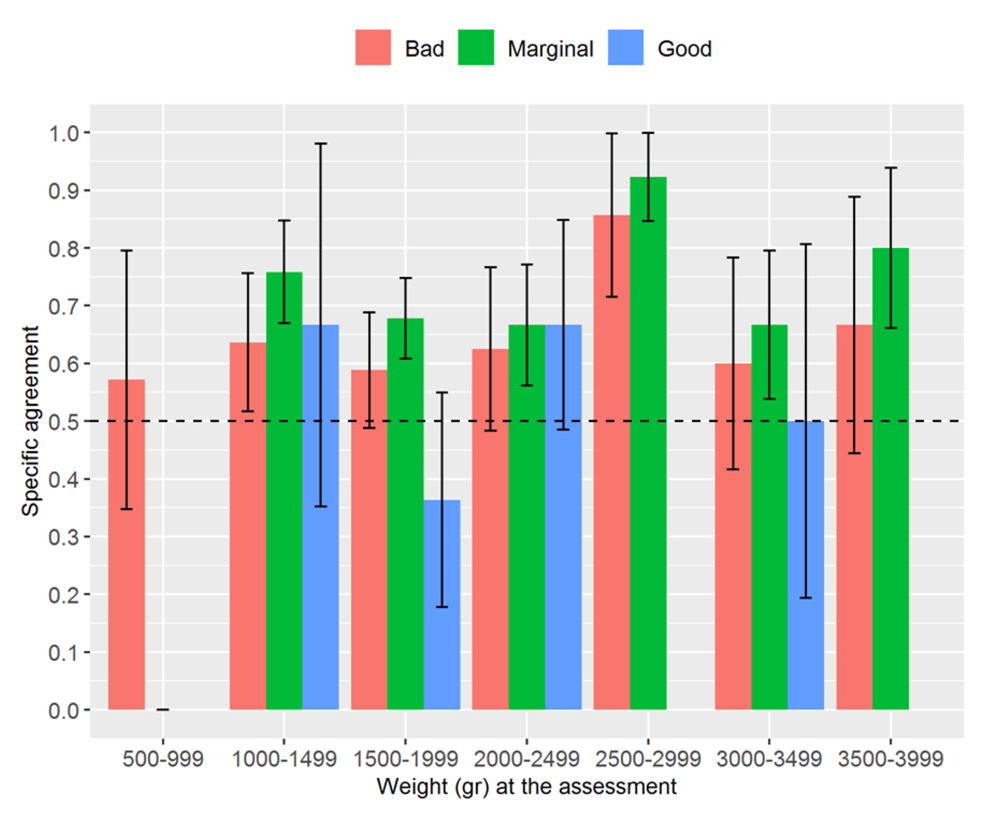


**Figure S2.** Proportion of specific agreements for categorical NAME grouped by weight at assessment. No one received a “Good” score in the groups 500-999 gr, 2500-2999 gr and 3500-3999 gr.
